# The SARS-CoV-2 mutation landscape is shaped before replication starts

**DOI:** 10.1590/1678-4685-GMB-2023-0005

**Published:** 2023-06-19

**Authors:** Diego Masone, Maria Soledad Alvarez, Luis Mariano Polo

**Affiliations:** 1 Consejo Nacional de Investigaciones Científicas y Técnicas (CONICET), Universidad Nacional de Cuyo (UNCuyo), Instituto de Histología y Embriología de Mendoza (IHEM), Mendoza, Argentina.; 2 Universidad Nacional de Cuyo (UNCuyo), Facultad de Ingeniería, Mendoza, Argentina.; 3 Consejo Nacional de Investigaciones Científicas y Técnicas (CONICET), Universidad Nacional de Cuyo (UNCuyo), Instituto de Medicina y Biología Experimental de Cuyo (IMBECU), Mendoza, Argentina.

**Keywords:** SBS spectra, viral replication niche, COVID19 vaccination status

## Abstract

Mutation landscapes and signatures have been thoroughly studied in SARS-CoV-2. Here, we analyse those patterns and link their changes to the viral replication tissue in the respiratory tract. Surprisingly, a substantial difference in those patterns is observed in samples from vaccinated patients. Hence, we propose a model to explain where those mutations could originate during the replication cycle.

Modifications in the mutation landscape of a genomic sequence can result through several mechanisms (Kucab et al., 2019), such as error-prone polymerases, metabolism, and damaging agents, as an unbalanced redox environment. The comprehensive analysis of the SARS-CoV-2 interhost single base substitution (SBS) showed a mutational spectrum dominated by C>U and, surprisingly, G>U substitutions (Di Giorgio et al., 2020; Panchin and Panchin, 2020; Popa et al., 2020; De Maio et al., 2021). Here we extend those studies to elucidate the impact of the replication niche and vaccination status on that pattern.

The SBS spectrum of SARS-CoV-2 from patients infected with alpha and delta variants was calculated (see methods in S1), confirming that it is dominated by C>U and G>U substitutions, followed by G>A and A>G (Figure 1A). Transition-type SBS -the interchanges between purines (C>U and U>C) or pyrimidines (G>A and A>G)- were expected to be the most frequent, as they can result from the activity of antiviral enzymes such as APOBEC and ADAR deaminases in host cells (Di Giorgio et al., 2020; Liu et al., 2021; Li *et al.*, 2022). In contrast, G>U transversions, particularly prevalent in SARS-CoV-2 (Forni et al., 2022), can result from stochastic processes, such as the misincorporation of nucleotides by an error-prone polymerase with a specific bias or the chemical modification of RNA. Those hypotheses have been discussed previously (Panchin and Panchin, 2020; De Maio et al., 2021; Mourier et al., 2021; Rice et al., 2021), and it is widely agreed that G>U transversion is caused by mutagen exposure, like oxidation due to reactive oxygen species (ROS). The process begins with the oxidation of a guanine base to produce 8-oxoguanine (8-oxoG). Like guanine, 8-oxoG can pair with cytosine; however, it can also pair with adenine (Figure 1B). Exceptionally, if 8-oxoG pairs with adenine during the first cycle of viral RNA replication, it can be substituted by uracil in the second replication cycle (Graudenzi et al., 2021). 

**Figure 1 -:**
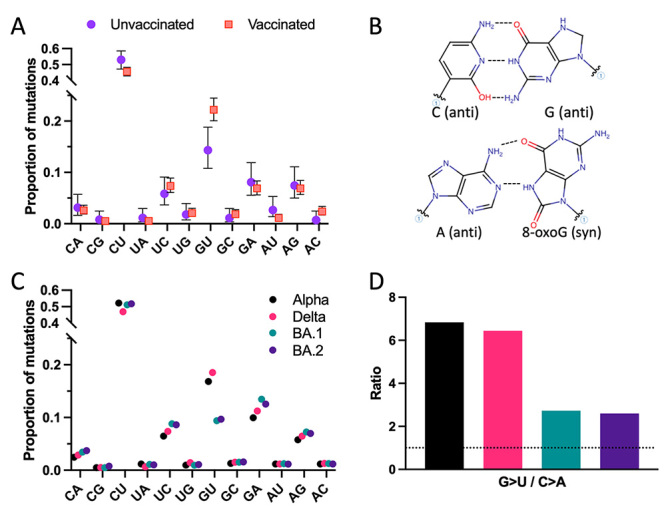
The environment of infected cells alters the G>U substitution incidence in SARS-CoV-2. (A) Analysis on SBS proportions across alpha and delta variants from unvaccinated (samples collected worldwide before 15th of January 2021, when less than 0.5% of the world population was vaccinated) and vaccinated patients. (B) Diagrammatic representation of standard (Watson and Crick) pairing of guanine and cytosine (top panel) and Hoogsteen base pairing between 8-oxoguanine and adenine (bottom panel). (C) Comparison of SBS spectra of different variants from vaccinated patients. Error bars denote confidence intervals (CI). (D) Proportion of G>U and C>A within the variants, coloured as in (C). The dotted line denotes no asymmetry (ratio=1).

Here, we considered how that misincorporation could occur during the intracellular life cycle of SARS-CoV-2. Various external mechanisms can explain modifications in the redox balance in infected cells, with the immune system as the prime suspect (Laforge et al., 2020). Therefore, if the immune system were indeed responsible for the changes in the oxidative environment of the infected cells (Laforge *et al.*, 2020; Paludan and Mogensen, 2022), differences would be expected between SBS spectra from unvaccinated and vaccinated patients (Collier et al., 2021; Szczepanek et al., 2022). Thus, we analysed samples from patients infected with alpha and delta variants divided into unvaccinated and vaccinated groups. Remarkably, G>U transversion was significantly altered (Figure 1A), sustaining a possible role of the immunological responses on the oxidative nature of those mutations. Moreover, the immune cells and those regulating their functions vary through the respiratory tract (Boers et al., 1998; Boers *et al.*, 1999). Thus, SBS patterns from those lineages infecting only part of the respiratory tract should differ from those that can infect the whole tract. For example, omicron subvariants (BA.1 and BA.2) mainly replicate in the upper respiratory tract (Meng et al., 2022), which reflects in a significant decrease in the G>U/C>A ratio when compared to alpha and delta, which can also replicate in the lower respiratory tract (Figure 1C,D). 

We hypothesised two scenarios where the nucleotide mispairing could occur when viral RNA (vRNA) is outside or inside double-membrane vesicles (DMVs), leading to different substitution patterns (Figure 2A,B). SARS-CoV-2 contains a positive non-segmented RNA genome [(+)vRNA]. Its replication comprises the early translation of a large polypeptide, then cleaved to produce the RNA-dependent RNA polymerase (RdRp). Both (+)vRNA and RdRp are compartmentalised into DMVs (Klein et al., 2020), avoiding the action of nucleases during vRNA replication (Mendonça et al., 2021). vRNA is then processed through double-stranded RNA intermediates in a sophisticated manner involving (+)vRNA and (-)vRNA. Nevertheless, some vRNA molecules generated inside DMVs are transported to the cytoplasm to produce viral structural proteins. In this scenario, G>U and C>A should have similar magnitudes if the mispairing occurs during replication inside DMVs (Figure 2A). However, G>U substitutions prevail over C>A (Figure 1A,C,D), favouring the theory where the mispairing happens before the vRNA is enclosed into a DMV (Figure 2B). Subsequently, the asymmetry between G>U and C>A transversions can be explained by inferring that guanine oxidation occurs mainly outside DMVs (Figure 2A), so compartmentalisation can play a role in decreasing the exposure of vRNA to the oxidative environment, protecting it from ROS action. Other two pairs of substitutions show asymmetry in their patterns, G>C/C>G and G>U/C>A (Figure 2C). The first of those pairs can be the product of ROS effect over guanine yielding imidazolone (Kino and Sugiyama, 2001), and the second one (G>U/G>A) could be mainly produced by the enzymatic activity of antiviral systems, as uracil is the outcome of cytidine deamination. Therefore, both asymmetries are explained by the protective role of compartmentalisation of the replicative machinery into DMVs. Remarkably, other coronaviruses shield their replication processes and machinery using DMVs (Miller and Krijnse-Locker, 2008). Consequently, it is unsurprising that the unbalance between those pairs of substitutions was also observed in MERS-CoV (Di Giorgio et al., 2020). 

**Figure 2 -:**
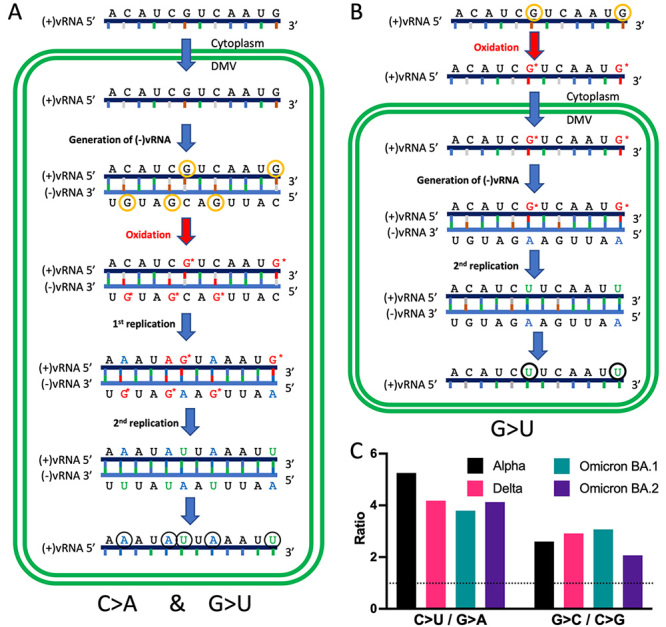
The influence of compartmentalisation on SBS patterns generated by oxidation. Differential pattern caused by mutagen exposure of vRNA guanines inside (A) or outside (B) double-membrane vesicles (DMVs), where vRNA replicates. Nucleotides circled in orange denote mutations that will occur in that scenario, while those in black mark the final product of the process. DMVs are delimited by double green lines. G* indicates 8-oxoguanine. (C) Proportion of C>U/G>A and G>C/C>G within the variants, coloured as in Figure 1C.

Additional studies are needed to elucidate in detail the mechanisms driving viral mutation patterns and how that drives the evolution of new SARS-CoV-2 strains. Particularly, if vaccines could cause novel strains appearance or to affect viral fitness through those mutations, further investigations are warranted to uncover how to manipulate that effect favouring their efficacy. 

## Supplementary material

The following online material is available for this article:

Data S1 - Material and methods
