# Supplementary material for: The SARS-CoV-2 mutation landscape is shaped before replication starts
Source: Genet Mol Biol. 2023 Jun 19;46(2):e20230005. doi: 10.1590/1678-4685-GMB-2023-0005 (PMC10280802; doi:10.1590/1678-4685-GMB-2023-0005)
Supplement: Data S1 - [file 1415-4757-GMB-46-2-e20230005-s1.pdf]

Genetics and Molecular Biology
https://doi.org/10.1590/1678-4685-GMB-2023-0005

Supplementary Material to "The SARS-CoV-2 mutation landscape is shaped before replication starts"

Data S1 – Material and methods

Calculation of SBS spectra

SBS mutational spectra for SARS-CoV-2 lineages were calculated using the 29th of July 2022 UShER SARS-CoV-2 phylogenetic tree (Turakhia *et al.*, 2021), as described in (Ruis *et al.*, 2022), with the small modifications described below.

To calculate SBS spectra for each vaccination status, we identified mutations on tip phylogenetic branches leading to sequences with known vaccination status in their GISAID metadata.

We calculated confidence intervals for mutation type proportions through the Wilson score interval using the total number of mutations as the number of trials and the proportion of the mutation type as the success proportion.

Data availability

The findings of this study are based on metadata available on GISAID (Khare *et al.*, 2021), via https://www.epicov.org/epi3/epi_set/EPI_SET_220927fu and https://www.epicov.org/epi3/epi_set/EPI_SET_220925np, for unvaccinated and vaccinated patients (respectively).

References

Khare S, Gurry C, Freitas L, Schultz MB, Bach G, Diallo A, Akite N, Ho J, Lee RT, Yeo W *et al.* (2021) GISAID's role in pandemic response. China CDC Wkly 3:1049-1051.

Ruis C, Peacock TP, Polo LM, Masone D, Soledad Alvarez M, Hinrichs AS, Turakhia Y, Ye C, McBroome J, Corbett-Detig R *et al.* (2022) Mutational spectra distinguish SARS-CoV-2 replication niches. bioRxiv. DOI:10.1101/2022.09.27.509649.

Turakhia Y, Thornlow B, Hinrichs AS, De Maio N, Gozashti L, Lanfear R, Haussler D and Corbett-Detig R (2021) Ultrafast Sample placement on Existing tRees (UShER) enables real-time phylogenetics for the SARS-CoV-2 pandemic. Nat Genet 53:809-816.
